# Accessible introductory exercises to crystallography databases and basic practices for undergraduate students

**DOI:** 10.1107/S2056989026006651

**Published:** 2026-06-30

**Authors:** Dylan J. Webb, René T. Boeré, Emily L. Trew, Jarrett O. Hanearin-Balczer, Elise A. Bennett

**Affiliations:** a Department of Chemistry & Physics, Mount Royal University, Calgary, Alberta T3E 6K6, Canada; b Department of Chemistry & Biochemistry, University of Lethbridge, Lethbridge, Alberta T1K 3M4, Canada; Harvard University, USA

**Keywords:** education, exercises, databases, undergraduate.

## Abstract

To adequately prepare and expose undergraduate students to practical crystallography, a set of exercises were designed to showcase the acquisition of data and information for a series of small mol­ecules. This dry-lab was made to be accessible and approachable irrespective of background knowledge on crystallography. Student commentary indicated a desire to learn more about crystallography where the main criticism was around downloading the software *Mercury*. Inclusion of these exercises did not require any redesign of the laboratory component of the course it was tested in. This work inspires further development of exercises to continue presenting crystallographic data practices to students at the undergraduate level.

## Abstract 2

The description and commentary for a set of simple exercises intended for undergraduate learners. These exercises introduce some basic practices for handling and reporting crystallographic data of small mol­ecules.

## Introduction

With the use of crystallography being commonplace in many fields and disciplines of chemistry, including but not limited to, coordination complexes with organometallic chemistry, proteins and biological materials with biochemistry, drug discovery and development, materials and minerals, small mol­ecule determination (Maveyraud & Mourey, 2020 ▸; Bijak *et al.*, 2023 ▸) and is the feature of several Nobel prizes (Jaskolski *et al.*, 2014 ▸; Galli, 2014 ▸): it is imperative that its practice is further integrated into an undergraduate university chemistry program. The practical component of crystallography is often found in teaching laboratories where crystallizing compounds is commonplace within several sub-disciplines of chemistry, but we rarely see usage of the data that can be produced from single crystals and why it is important. What makes learning about crystallography data prohibitive is access to resources, namely an X-ray diffractometer. Without a diffractometer available, there is often little motivation to teach crystallography data at an appreciable level despite the aforementioned disciplines being readily taught in a typical chemistry program curriculum (Halford, 2016 ▸). Theory can be included in spectroscopy courses or in graduate courses, but seldom do we see practical exercises implemented at the undergraduate level without substantial re-design of existing courses or the requirement of the cost-prohibitive instrumentation.

With a desire to expose undergraduate students to research in the field of chemistry, but being limited in resources available, there was motivation to produce a set of exercises that does not restrict any institution from including crystallographic databases and practices into their program or laboratory component. Where these exercises will require access to inter­net and computers, as is standard with crystallographic data analysis, institutions have these available and all the associated programs and data are freely accessible. A key component to operating with crystallographic data is knowing the programs and databases where the data are made available. It is unlikely that the practice of using the program *Mercury* (Macrae *et al.*, 2020 ▸) and accessing data from the Cambridge Structural Database (CSD; Groom *et al.*, 2016 ▸) to submit and access crystallographic data is lessening; therefore, to introduce crystallography databases and practices to undergraduate students should involve them.

While there are paid versions of programs and software that can provide in-depth learning modules (Brannon *et al.*, 2020 ▸), the exercises in this article were designed where access to these is not readily possible. The CSD is a database that can provide a myriad of learning opportunities, as demonstrated in the literature (Abourahma, 2024 ▸), and using data available is a fantastic avenue to introducing crystallography into a class from high school and above (Irmer, 2025 ▸). The current motivation is for short exercises that complement a synthetic laboratory component.

There are exercises involving crystallography for teaching laboratories which involve the structure determination of student generated materials with appropriate software [*SHELX* (Sheldrick, 2015 ▸) and *Olex2* (Dolomanov *et al.*, 2009 ▸)] complemented with other modules (synthesis and three-dimensional printed models) (Brannon *et al.*, 2020 ▸; Abrahams *et al.*, 2023 ▸). The aspects of structure determination are important to the discipline of crystallography and are best taught when supplemented with the course component as fundamentals need to be better introduced and with more dedicated laboratory sessions. Where there are time constraints and the theory is not strictly covered to a high degree, it can be challenging to incorporate appropriate exercises into a chemistry program.

Reported exercises use the necessary X-ray diffraction instrumentation, learning more about the process of selecting and mounting the single crystals, followed by data collection and refinement with aid from a crystallographer (Wilson *et al.*, 2012 ▸; Zheng & Campbell, 2018 ▸). This is not always available or accessible and is highly dependent on the institution. There are similar exercises that provide modality to experiments to function with those institutions lacking instrumentation access, allowing for students to grow their own organic single crystals suitable for data collection but where the collection itself may take place offsite (Bazley *et al.*, 2018 ▸). This is an extremely attractive approach to be adapted to Mount Royal University (MRU) if future course and laboratory time allows as well as access to instrumentation at other institutions. Initiatives regarding outreach to foster hands-on learning with other institutions would alleviate the lack of instrumentation (Zheng *et al.*, 2025 ▸), a consideration for future iterations if the appetite from learners is demonstrated.

The Cambridge Crystallographic Data Centre (CCDC), which provides the CSD, has detailed activities and learning modules regarding the navigation of their software and databases (Battle *et al.*, 2010*a* ▸,*b* ▸, 2011 ▸; Battle & Allen, 2012 ▸). While these are well described exercises with valuable learning objectives, their application would be better suited to a dedicated crystallography course with more devoted dry-lab time. Other similar resources include the class activities produced by chemistry education users in the *IonicViper* community (https://www.ionicviper.org/). Where the submitted exercises on *IonicViper* can be adopted as dry-lab components due to their user-friendly reporting, the motivation with the work presented in this article came from its relevance to the practical component.

**Figure 1:**
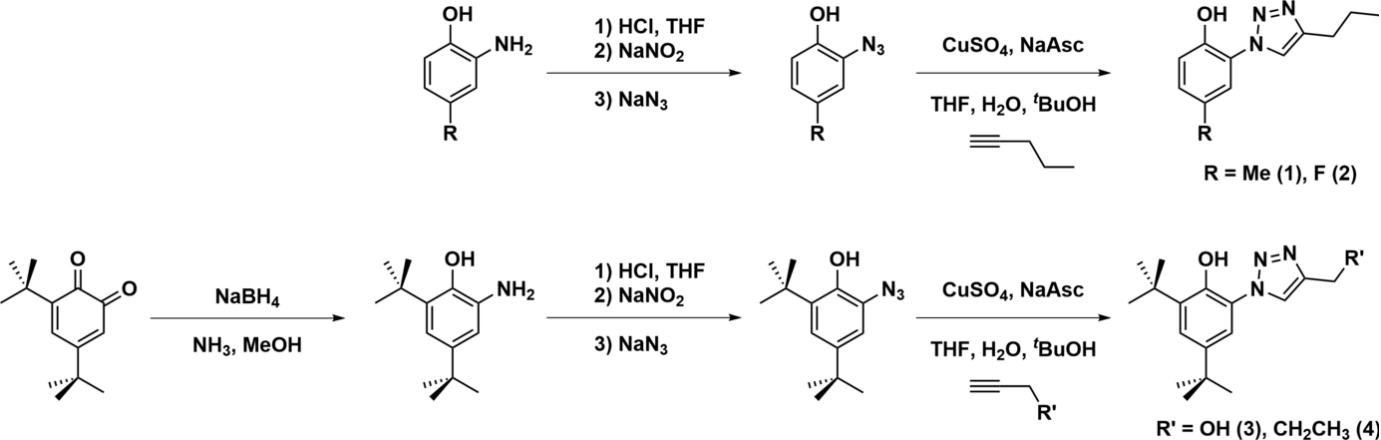
The synthesis of compounds **1**–**4** as part of the wet-lab portion not discussed in this article.

**Figure 2:**
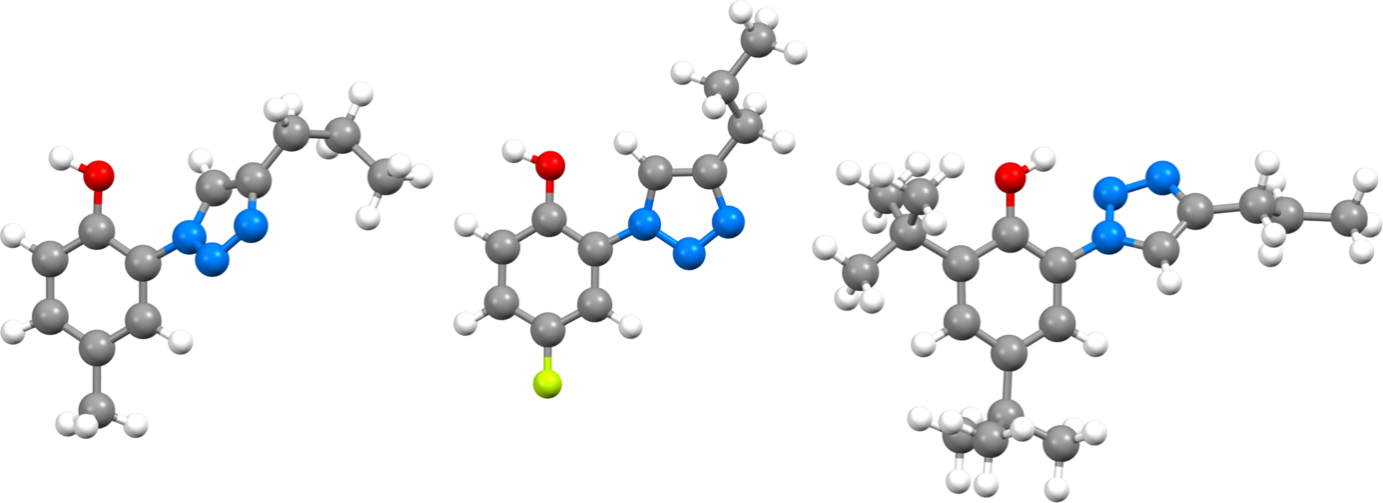
Ball-and-stick depictions (generated in *Mercury-CSD*, release 2025.3; Macrae *et al.*, 2020 ▸) of three mol­ecular structures of compounds **1**–**3** (left to right) from single-crystal structure determinations. These compounds were targets of the wet-lab procedure and their models used in the dry-lab exercises.

From the development of the dry-lab exercises in coordination with the wet-lab component, it became apparent that they presented a low-threshold access to working with the CSD and the software *Mercury* where the student experience could be collected alongside the wet-lab component that was already underway. The practical, or wet-lab, component is the feature of a separate article. This separate article focuses on the assessment of practical laboratories with a novel approach to evaluating competency and therefore these articles present different contributions to the literature. The following is the synthesis of four compounds that have been adapted for use in an undergraduate teaching laboratory (Fig. 1 ▸). The compounds 2-(4-propyl-1*H*-1,2,3-triazol-1-yl)-4-methyl­phenol, (**1**), 2-(4-propyl-1*H*-1,2,3-triazol-1-yl)-4-fluoro­phenol, (**2**), and 2,4-di-*tert*-butyl-6-(4-propyl-1*H*-1,2,3-triazol-1-yl)phenol, (**3**) crystallized to produce high-quality single crystals, the data of which are the subject of this article (Fig. 2 ▸); whereas 2,4-di-*tert*-butyl-6-[4-(hy­droxy­meth­yl)-1*H*-1,2,3-tri­azol-1-yl]phenol (**4**) did not crystallize sufficiently and is part of a learning outcome in the other article. The synthesis of these compounds came from a research project with asymmetric ligands with triazole groups and is the focus of future publications.

To complement the practical component and introduce crystallographic data to learners, the dry-lab component was developed, which is presented in this article. The aim was to have a dry-lab component run simultaneously with the wet-lab without the need for additional lab time; and in conjunction with the wet-lab to complement the learning experience. This way, when crystals of compounds **1**–**3** are obtained by the learners as part of the practical exercises, the learners can work with the relevant data they would have acquired were a diffractometer available. The learners therefore get a more research relevant experience working in a synthetic chemistry laboratory. The dry-lab component is presented and discussed along with insight into the student experience from their first introduction to crystallography. Skills developed in these exercises include (i) analysis and compilation of geometrical data, (ii) identification of hydrogen bonding, and (iii) categorization of hydrogen bonding using Etter (1990 ▸) notation.

## Teaching exercises

The laboratory component for *CHEM4411 – Organometallic & Catalysis* at MRU was designed around the synthesis of a series of new compounds of relevance to the discipline. The original syntheses were undertaken by undergraduate researchers at MRU. During characterization, the products were also grown as single crystals suitable for single-crystal X-ray crystallography (SCXRD). Crystallographic data were collected, solved and refined at the University of Lethbridge and the data were submitted as Experimental Crystal Structure Determinations (with individual DOIs) to the CSD with the appropriate co-authorships (Webb *et al.*, 2025*a* ▸,*b* ▸,*c* ▸). A series of exercises were created with the intention of allowing future students enrolled in the course to benefit from structure analysis of their laboratory products in an environment where a diffraction facility was not readily available. These structures were ‘real life’ examples, chosen because they included some of the synthetic targets of the wet-lab portion. The structures were screened for having sufficient crystal model quality to make their treatment tractable to beginners and suitable for extracting geometric information. All are phenolic triazoles and display (different kinds of) inter­molecular inter­actions via hydrogen bonding. Nevertheless, they display the range of structural outcomes expected from actual structure determinations in organic and coordination chemistry. For example, the structure of **1** includes a disordered *n*-propyl side chain, whereas the same kind of side chain in **2** and **3** are not disordered.

The exercises were then adapted for use at MRU in the Winter 2026 iteration of *CHEM4411* (enrollment = 8). The learning outcomes for this dry-lab were optimized to the specific nature of the crystallographic exercises, which was separated from the wet-lab. These dry-lab exercises, their objectives, and the student experience are detailed in this article. Other courses within the chemistry program at MRU were not suitable for the inclusion and testing of these exercises as the authors were not able to alter other laboratory sections at this time.

Students in the *CHEM4411* course are considered senior level chemistry students at MRU. The prerequisites include introductory inorganic chemistry and the second organic chemistry course (at MRU, these are *CHEM2401* and *CHEM2102*). The students have basic laboratory skills and are well aware of the typical procedures used in practical teaching laboratories. By the time they reach the 4000 level courses, students in the chemistry program at MRU have experience with spectroscopy, usually having taken the spectroscopy course or have been exposed to the techniques in the laboratory components of other science courses. It should be noted that the students have no experience with crystallography before *CHEM4411*, and have only been exposed to the concept through discussions of the practical applications (such as protein crystallography and visualization in biochemistry courses along with concepts of symmetry and related classifications). Therefore, the exercises presented in this article serve as a complete introduction to the concept of crystallography along with the practical application of the data that complements the generation of the relevant crystals in the wet-lab portion (see Fig. 1 ▸ for details). The dry-lab exercises (see Appendix A in the supporting information) were provided to the learners when the wet-lab portion began in the semester, are fully integrated into the lab manual for this course, and could be completed at any time over the latter six weeks of the Winter semester. Computers and associated resources were available on campus, but the exercises could also be completed off-campus on personal computers. This was to provide an accommodating approach and ample time for the students to complete these exercises. The aim was for the exercises to be completed within a four-hour time period, the standard laboratory time for *CHEM4411*. Extra time in the semester for the laboratory component of* CHEM4411* was made available for the students, ensuring that there was dedicated time to complete the dry-lab. The exercises here were chosen to appropriately demonstrate use of the CSD and the program *Mercury* by beginners. With basic computer skill competency, the learners can demonstrate several learning outcomes:

**·** Tabulation of important crystallographic information (bond distances, angles, and torsions) while referencing standard values.

**·** Common phenomena observed with crystallographic information (disorder, hydrogen-bonding structures/networks)

**·** Accessing the Cambridge Crystallographic Data Centre website (https://www.ccdc.cam.ac.uk/ for downloading software and accessing data from the CSD)

**·** Use of basic commands with the program *Mercury* (labelling, display options, data tabulation, structure visualization)

**·** Introduction to Etter notation for hydrogen bonding

All these outcomes are spread through the eight report questions (see Appendices A and B in the supporting information), which incorporate opportunities to compare and contrast information between the three compounds – data comparison being common practice for crystallographers.

## Student Experience

In order to include the student experience and disseminate comments regarding this procedure and how the competency determination was handled, feedback was requested from the students participating in the Winter 2026 iteration of *CHEM4411 – Organometallic & Catalysis*. Ethics approval to use student feedback was obtained: MRU Human Research Ethics Board (HREB) application ID No. 104745. Consent was obtained from student participants at the beginning of the semester by a member of the research team, who maintained the confidentially of the study participants until after the grade appeal deadline. This was to manage conflict of inter­est that would arise from students who were or were not participating, allowing all students to be assessed equally. Optional additional feedback was available through an anonymous online survey that was shared when the final grades for the laboratory component were made available (see Appendix C in the supporting information). Students were informed on the purpose of the experiment, the dissemination of the information, and what the assessment for this experiment entailed. It should be noted that the survey questions for the student experience were not designed to assess these learning objectives, but instead to provide insight into the accessibility of the exercises, how well the dry-lab complemented the wet-lab, and the appetite for further inclusion of crystallography into the curriculum.

### Student Feedback

The student experience was positive. With students agreeing that they produced an understanding of crystallography and more agreeing that this dry-lab made them curious about crystallography as a subject. The accessibility of this dry-lab came with a neutral response along with a desire for a longer lab involving crystallography. Most learners agreed that this dry-lab complemented the wet-lab that run concurrently in the semester.

Positive comments included:

**·** ‘Overall, we learned a lot. It was inter­esting to use the software to explore the crystal structures and actually visualize how everything is arranged. This helped us better understand what we were doing in the wet lab, so the dry lab complemented it really well.’

**·** ‘… it was a nice dry lab and helped expand my knowledge on crystallography which I was not familiar with before.’

Critical comments included:

**·** ‘We spent a lot of time downloading and setting it up instead of doing the lab. It would be better if it was already installed on the CHEM lab computers so we can focus more on the lab itself.’

**·** ‘It was just difficult to get hands on the software as it was took too much storage and would stop while downloading in many computers and laptops but some computers were compatible and it worked out.’

**·** ‘Having a class where learning how to use the software would have been beneficial. Learning more about crystallography before would have also been helpful.’

## Discussion

From the perspective of the instructor, the students had few issues completing the exercises and there were minimal questions regarding the content overall. With each of the eight report questions split into three exercises (one for each of the compounds used), reports produced were all completed to a high degree (see Appendix C in the supporting information). For exercise 1, report questions introduced disorder, tabulation of data crystallographic data, hydrogen bonding, and inter­molecular connections with the Etter notation. This exercise proved to be the most difficult with students losing marks with their understanding of disorder. As this was the place where they learned about disorder for the first time, the explanations were somewhat weak for a senior level student. Concepts of this being introduced elsewhere would bolster the answers given. For exercises 2 and 3, where report questions repeated similar concepts with different examples, the students performed well with issues coming from how the screenshots of the mol­ecules were generated or their quality along with the overall depth of questions. As this was a senior level class, the expectations were higher than if these exercises were given to learners from earlier years.

From the feedback, the main issue learners identified was that the program, *Mercury*, was quite large and took a considerable amount of time to download or that the on-campus computers needed administrative permission for the download to occur. With planning, this is an issue that can be easily circumvented and does not represent student ineptitude with the exercises. Other issues were mostly with students not reading all the instructions before beginning each exercise, something that only few students verbally commented on. The repetitive nature of the exercises was commented on; while an understandable criticism it should be noted that repetition was included to reinforce the learning outcomes (Wiggins *et al.*, 2021 ▸). Having more class time dedicated to the some basic concepts of mol­ecular crystallography would likely enhance the experience and will be considered in future planning.

The success of this dry-lab, with how it complements the wet-lab – something the learners agreed on – means that these exercises will be implemented into future iterations of *CHEM4411*. To remedy issues with understanding crystallography concepts, related material will be introduced in the course or prerequisites where appropriate (such as disorder and collection methods). Where the laboratory component for this course encompasses many aspects of synthesis, these exercises successfully acknowledge and introduce characterization that comes from obtaining single crystals and the associated data. With the use of crystallographic data being commonplace in organometallic chemistry, small mol­ecule determination, and protein research, it is imperative that these techniques are introduced to some capacity during related undergraduate programs regardless of the presence or access to the instrumentation – which are often unobtainable at primarily undergraduate institutions. The programs and data for these exercises are all currently freely available, making it accessible to any institution wanting to leverage some tested exercises for their own programs.

Allowing students to complete dry-labs at their own pace, manage their own time, all while providing space and time to complete it on campus, makes it an effective addition to the laboratory component. This article is meant to be an example of such an exercise that can be adapted as needed and acts as a quick, reliable, and accessible dry-lab to introduce the basic practices and databases used in crystallography for an undergraduate laboratory. We agree with a Reviewer criticism that starting the exercises with compound **1** may have been unnecessarily complicated as it is the sole example exhibiting a positional disorder. A possible improvement might be to renumber the exercises such that compounds **2** and **3** are encountered first (with relabelling) or replacing compound **1** by a similar type of synthetic target that yields an ordered crystal structure. However, probably an even more helpful improvement will be to add some specific support on special features (such as this disorder) within the Exercises text. We note that the specific ability of *Mercury* to filter out minor components of structural disorder provides a sufficient remedy for managing the Exercises – so long as sufficient explanation of the phenomenon is included. Along with the next iterations taking into account of the learners here, more exercises are being developed with these data. Future directions from this work involve exercises that use other freely available software for structure refinement and navigating CIF files, all of which will need to be pre-installed to available computers for ease of accessibility.

## Supplementary Material

Appendices A, B, and C. DOI: 10.1107/S2056989026006651/oi2039sup2.docx

## Data Availability

As is the purpose of the article, all data and associated software are freely available online (at the time of publication) in the repositories and all links and information is provided throughout the article and the included exercises in the supporting material.
