# Supplementary material for: Accessible introductory exercises to crystallography databases and basic practices for undergraduate students
Source: Acta Crystallogr E Crystallogr Commun. 2026 Jun 30;82(Pt 7):893–8. doi: 10.1107/S2056989026006651 (PMC13330842; doi:10.1107/S2056989026006651)
Supplement: Supplementary file 1 [file e-82-00893-sup2.docx]

Supporting Information

Accessible Introductory Exercises to Crystallography Databases and Basic Practices for Undergraduate Students

Dylan J. Webb,^a^ René T. Boeré,^b^ Emily L. Trew,^b^ Jarrett O. Hanearin-Balczer,^a^ Elise A. Bennett^a^

^a^ Department of Chemistry & Physics, Mount Royal University, Calgary, Alberta T3E 6K6, Canada

^b^ Department of Chemistry & Biochemistry, University of Lethbridge, Lethbridge, Alberta T1K 3M4, Canada

Contents

[Appendix A: Lab Manual Example 2](#_Toc232161234)

[Appendix B: Report Questions and Some Example Answers 10](#_Toc232161235)

[Appendix C: Survey Questions and Results 18](#_Toc232161236)

# Appendix A: Lab Manual Example

**Crystallographic Investigation of Small Molecules**

*Exercise 1: Analysis of 3D-Geometrical Data for Compound 1*

Use of the sophisticated but user-friendly chemical crystallography software Mercury-CSD (hereafter “Mercury”) is required for this Exercise. If not already available on laboratory computers, it may be freely downloaded and installed from the web page: <https://www.ccdc.cam.ac.uk/support-and-resources/downloads/>, via the button “Free Mercury including enCIFer (for Non-Commercial use)” and selecting the relevant macOS, linux or Windows installers. *After completing the installation, open Mercury and in the window that opens first, select Activate CSD-Community*.

Now, download the data for the XRD structure of **1** using the DOI from reference 1. It is identified by the *CSD Refcode* HUHMIK. Press on Download and select Download current entry. This opens a window such as the one below – fill in your name and university email address and accept the terms and conditions. The data will be downloaded as a ZIP file, which must be extracted into a folder called 2417106, wherein is a file called 2417106.cif. This file is opened using the program Mercury; setting this as the default program for opening this type of file on your computer may expedite the work.


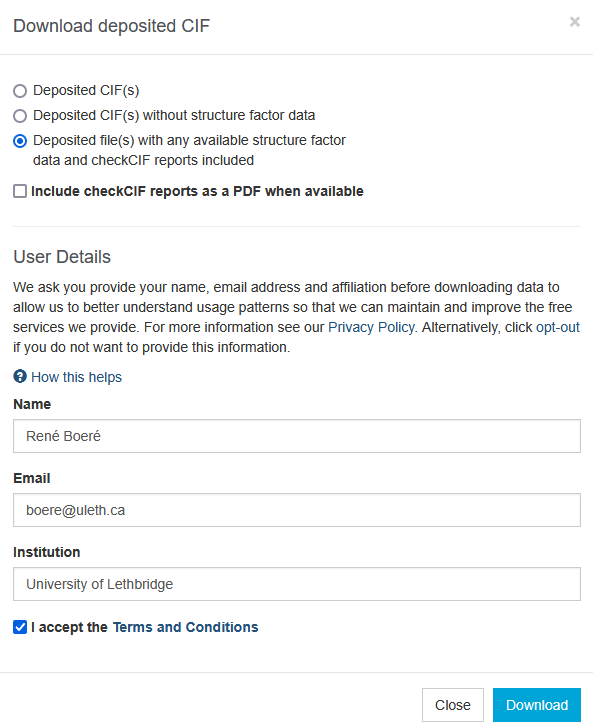


After opening the file, it is possible to customize the Mercury environment. For this exercise, the Style: *Ball and Stick* is recommended (use Display/Styles/Ball and Stick Settings to choose the view you like. A non-metal radius of 0.10 or 0.15 should work, as will a bond-radius of no more than 0.05.).

In the structure of **1**, the *n*-propyl substituent is *disordered* between two sites. An alternative that can also be recommended is to use *Ellipsoid* (likely the default in a new installation); if using this, then under Display/Styles/Ellipsoids Settings, uncheck the Draw hydrogens as fixed-size spheres options. Illustrations in this guide will employ this option, which has the advantage of an accurate representation of the vibrational motion of the hydrogen atoms. It is recommended that you simplify the analysis by selecting the main component of the disorder by pressing on “A” in the disorder bar near top right of the interface window:
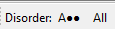
.

**REPORT QUESTION 1:** *Why is there disorder in the crystal structure? Comment on this and address why disorder is occurring for the n-propyl chain. In your answer, show a screenshot of both components (i.e. the two disordered components) which can be obtained in the Disorder button by pressing alternately on the left and right dots or circles.*

Further customize the display window using the Calculate tab, and selecting Contacts…


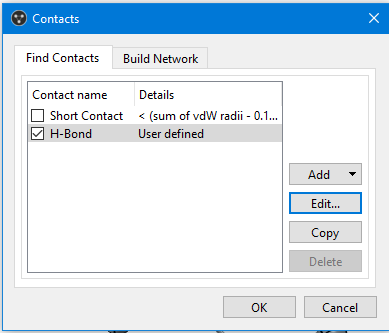

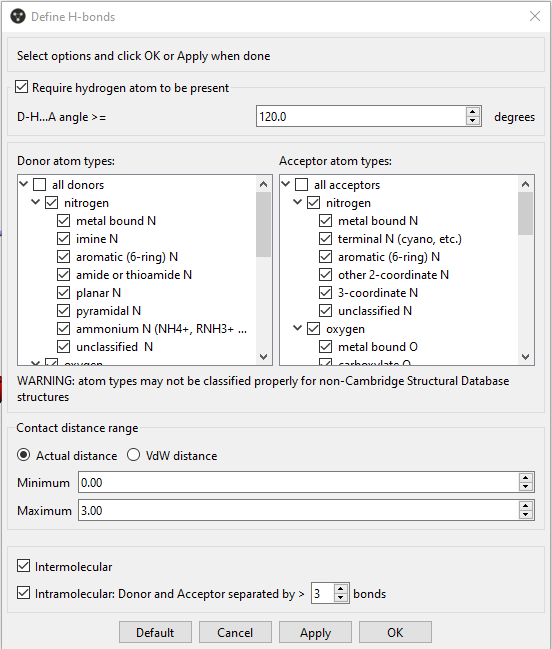


Within the small Contacts window, check off *only* H-bond, then *Edit* for options. In the larger Define H-bonds window, ensure *Require hydrogen to be present* is checked on. Additionally, since a conventional H-bond involving 2^nd^ period elements should never exceed 2.5 Å, you should ensure that the *Actual Distance* has a Maximum distance setting of 2.50 (see references 2 & 3).

*Molecular Geometry*

Under the *Display* drop down menu, under *Styles*, uncheck *Display Bond Types* and *Display Aromatic Rings*. You should be able to figure out and explain the bond types based on geometry around the atom, which you can determine shortly.

Use the *Picking Mode* in Mercury to select (sequentially) *Measure Distance*, then *Measure Angles*, and finally *Measure Torsions*. For each category, use mouse clicks on the non-H atoms to collate the bond distances in the **phenol** and **triazole** rings as well as the C–O bond length (click two sequential atoms). A Tabular format (as a Tab Separated Values spreadsheet-compatible file) can alternatively be obtained from Display/More Information/Distance (or, Angles, Torsions). For angles e.g. around the rings, click three sequential atoms, and for torsions four sequential atoms. For example, the torsion angles C1-C2-N1-N2 and C3-C2-N1-C7 provide a measure of the ‘relative rotation’ between the two rings. It is usually helpful to press Clear Measurements between measurement types to avoid undue clutter on your computer screen.

**REPORT QUESTION 2:** *Tabulate the information found from measuring the distance, angles, and torsions of the rings in compound 1.* *Discuss the geometry that you measure with reference to standard values expected (e.g. from textbooks or reference works) for substituted aromatic and heterocyclic compounds. Use of a spreadsheet to organize the compilation is a recommended option.*

*Hydrogen Bonding*

When you click and check off the H-Bond option in Calculate Contacts, Mercury will display the first (probe) atom of groups that are H-bonded to the principal molecule *and* the Picking Mode changes to Expand Contacts. Now, explore the possible H-bonds in your structure by clicking on these probe atoms, and continuing until you get a sense of the ‘hydrogen bonding pattern’ of structure **1**. Continue to click on the atoms to expand the pattern (*alternatively* under Contacts 🡪 Build Network tab, click on the Expand All button several times, speeding up the process).

If you ever get stuck, close the windows and select “Asymmetric unit” at the bottom left to return to a single molecule. Un-select it to continue working on the structure.

**REPORT QUESTION 3:** *Describe the hydrogen bonding structure observed. Consider the symmetry and shape of the observed structure. What may help, is considering that this compound is in what is called a space group labelled as P6_1_. In your answer, include a screenshot showing the structure.*

*Etter Notation*

Margaret C. Etter was a brilliant crystallographer and beloved chemistry teacher at the University of Minnesota who devised a consistent way to classify H-bonding patterns in molecular crystal structures (see reference 3 for details). The ‘Etter notation’ for the simple chain structure determined above is $C_{1}^{1}(7)$ – see reference 4, Table 1 and section 3, for definitions of graph set notation with illustrative examples – which designates that a chain is formed (hence *C*), with a single H-bond donor (the OH, shown by the subscript *1*) and a single acceptor (the N3 atom of the triazole, shown by the superscript *1*) and a *degree* of 7 (i.e. the shortest chain of atoms that are replicated in the chain is seven, N3-N2-N1-C2-C1-O1-H1).

**REPORT QUESTION 4:** *Show a screenshot of the shortest chain of atoms that creates this structure with labels. Use the “Display” followed by “Atom Labels” and select “Label by atom Label” and “Picked atom” to display and select labels. You can move these labels under the Picking mode “Move labels”.*

*Exercise 2: Analysis of 3D-Geometrical Data for Compound 2*

Download the data for the XRD structure of **2** using the DOI from reference 5. It is identified by the *CSD Refcode* HUHMOQ. Press on Download and select Download current entry. This opens a window such as the one below – fill in your name and university email address and accept the terms and conditions. The data will be downloaded as a ZIP file, which must be extracted into a folder called 2417107, wherein is a filed called 2417107.cif. This file is opened using Mercury.

*Molecular Geometry*

As described in the same section of Exercise 1, use the Picking Mode in Mercury to analyze the geometry of the structure of **2**.

**REPORT QUESTION 5:** *Are there any noteworthy differences between the measured geometries of* ***1*** *and* ***2****? Using torsion angles, comment on the relative orientations of the phenol and triazole rings in the two structures. Discuss the geometry that you measure with reference to standard values expected (e.g. from textbooks or reference works) for substituted aromatic and heterocyclic compounds.*

*Hydrogen Bonding and Etter Notation*

Select the *Calculate/Contacts*… option, and check on the H-bond setting (remember to ensure that the other box, i.e. Short Contact, is left *off*). Then *expand* the contacts as described in Exercise 1 to determine the nature of the extended H-bond pattern in this crystal lattice.

**REPORT QUESTION 6:** *Describe the hydrogen bonding structure observed. Consider the symmetry and shape of the observed structure. Compare and contrast this with compound* ***1****. What may help, is considering this compound is in what is called a space group labelled as P2_1_/c.*

*In your answer, include a screenshot showing the hydrogen bonding structure and another screenshot with atoms labeled showing the shortest chain of atoms that create this structure (like questions 3 & 4). What would the Etter notation be?*

*Exercise 3: Analysis of 3D-Geometrical Data for Compound 3*

Download the data for the XRD structure of **3** using the DOI from reference 6. It is identified by the *CSD Refcode* HUHMUW. Press on Download and select Download current entry. This opens a window such as the one below – fill in your name and university email address and accept the terms and conditions. The data will be downloaded as a ZIP file, which must be extracted into a folder called 2417109, wherein is a filed called 2417109.cif. This file is opened using Mercury.

*Molecular Geometry*

As described in the same section of Exercise 1, use the Picking Mode in Mercury to analyze the geometry of the structure of **3**.

**REPORT QUESTION 7:** *Are there any noteworthy differences between the measured geometries of* ***3*** *with the compounds? Using torsion angles, comment on the relative orientations of the phenol and triazole rings in the two structures. What range of ring-twisting angles are observed for this set of three similar chemical structures? Why? Discuss the geometry that you measure with reference to standard values expected (e.g. from textbooks or reference works) for substituted aromatic and heterocyclic compounds.*

*Hydrogen Bonding and Etter Notation*

Here too, select the Calculate/Contacts… option, and check on the H-bond setting.

This molecule adopts a very different kind of H-bonding pattern, here the two molecules form a *discrete dimer*. The need, the so-called *chemical potential*, that the H-bond donors O1 and H-bond acceptors N3 feel to share these H-atoms, an extended chain does not form (despite the fact that the crystal lattice symmetry of **3**, space group *P*2_1_/*n* is effectively the same as that in **2**). Considering other differences in the molecular structures of **1** and **2**, versus that of **3**, propose a rationale for the adoption of these two different patterns.

With this very different pattern, we might expect that the Etter classification also changes. Indeed, that is the case, there is first of all a new kind of classification, designated as $S_{1}^{1}(6)$. This defines an *intramolecular* hydrogen bond, still with a single H-bond donor (the OH, shown by the subscript *1*) and single acceptor (which is now the *N2 atom* of the triazole, shown by the superscript *1*) but the *degree* is 6 (i.e. the shortest chain of atoms is now: N2-N1-C2-C1-O1-H1).

But there is more! *Two of the molecules* of **3**, each of which has the intramolecular as $S_{1}^{1}(6)$ pattern, also come together in a pattern designated by the Etter notation $R_{2}^{2}(14)$. Here *R* designates the very evident ring pattern observed. Note that this now has two donors (i.e. the two O1 atoms) and two acceptors (i.e. the two N3 atoms). Where else do we observe N3 as an acceptor? Does that indicate a *molecular similarity* between similar molecular structures, despite a difference in overall H-bonding pattern? It is these similarities that Margaret Etter was determined to catalog in her efforts to globally categorize H-bonding in organic crystal lattices. The *degree* here is, however, very different, and is given as 14 atoms. Where do we see a ring of 14 atoms? Note that this takes the ‘long way around’, i.e. the link H1-O1-C1-C2-N1-N2-N3-H1-O1-C1-C2-N1-N2-N3. Specifically, there is no ‘short cut’ through the H1∙∙∙N2 path. It is important in the mathematics of *graph theory* on which the Etter notation is based, to not count the same interaction twice over.

**REPORT QUESTION 8:** *Describe the hydrogen bonding structure observed. Consider the symmetry and shape of the observed structure. Compare and contrast this with compounds* ***1*** *&* ***2****. What may help, is considering this compound is in what is called a space group labelled as P2_1_/n.*

*In your answer, include a screenshot showing the hydrogen bonding structure/dimer and another screenshot with atoms labeled showing the “long way around” link as described in the additional Etter notation above.*

*References*

1) Webb, D. J.; Boeré, R. T.; Trew, E. L. CCDC 2417106: Experimental Crystal Structure Determination, 2025. https://doi.org/10.5517/ccdc.csd.cc2m4655.

2) Boeré, R. T. Hydrogen Bonds Stabilize Chloroselenite Anions: Crystal Structure of a New Salt and Donor-Acceptor Bonding to SeO2. *Molecules* **2023**, *28* (22), 7489.

3) Bernstein, J.; Davis, R. E.; Shimoni, L.; Chang, N-L. Patterns in Hydrogen Bonding: Functionality and Graph Set Analysis in Crystals. *Angew. Chem Int. Ed. Engl.* **1995**, *34* (15), 1555-1573.

4) Etter, M. C. Encoding and decoding hydrogen-bond patterns of organic compounds. *Acc. Chem. Res.* **1990**, *23* (4), 120-126.

5) Webb, D. J.; Boeré, R. T.; Mounce-McKinney, K. J. CCDC 2417107: Experimental Crystal Structure Determination, 2025. https://doi.org/10.5517/ccdc.csd.cc2m4666.

6) Webb, D. J.; Boeré, R. T.; Hanearin-Balczer, J. O. CCDC 2417109: Experimental Crystal Structure Determination, 2025. https://doi.org/10.5517/ccdc.csd.cc2m4688.

# Appendix B: Report Questions and Some Example Answers

*REPORT QUESTION 1: Why is there disorder in the crystal structure? Comment on this and address why disorder is occurring for the n-propyl chain. In your answer, show a screenshot of both components (i.e. the two disordered components) which can be obtained in the Disorder button by pressing alternately on the left and right dots or circles.*

*REPORT QUESTION 2: Tabulate the information found from measuring the distance, angles, and torsions of the rings in compound 1. Discuss the geometry that you measure with reference to standard values expected (e.g. from textbooks or reference works) for substituted aromatic and heterocyclic compounds. Use of a spreadsheet to organize the compilation is a recommended option.*

**NOTE**: Learners provided tabulated data for all three requested pieces of information. Several learners also provided an image, like this above, with the data they tabulated (not shown in this appendix for space).

*REPORT QUESTION 3: Describe the hydrogen bonding structure observed. Consider the symmetry and shape of the observed structure. What may help, is considering that this compound is in what is called a space group labelled as P61. In your answer, include a screenshot showing the structure.*

**NOTE**: Given crystallography is introduced as part of this dry-lab, the language used in several answers, like this above, do not reflect typical nomenclature used in the discipline.

*REPORT QUESTION 4: Show a screenshot of the shortest chain of atoms that creates this structure with labels. Use the “Display” followed by “Atom Labels” and select “Label by atom Label” and “Picked atom” to display and select labels. You can move these labels under the Picking mode “Move labels”.*

**Grading average for Exercise 1 (report questions 1-4): 86%**

*REPORT QUESTION 5: Are there any noteworthy differences between the measured geometries of 1 and 2? Using torsion angles, comment on the relative orientations of the phenol and triazole rings in the two structures. Discuss the geometry that you measure with reference to standard values expected (e.g. from textbooks or reference works) for substituted aromatic and heterocyclic compounds.*

**NOTE**: Figure and data tabulation is similar to that of *REPORT QUESTION 2*

**

**

*REPORT QUESTION 6: Describe the hydrogen bonding structure observed. Consider the symmetry and shape of the observed structure. Compare and contrast this with compound 1. What may help, is considering this compound is in what is called a space group labelled as P21/c.*

*In your answer, include a screenshot showing the hydrogen bonding structure and another screenshot with atoms labelled showing the shortest chain of atoms that create this structure (like questions 3 & 4). What would the Etter notation be?*

**

**

**Grading average for Exercise 2 (report questions 5-6): 96%**

*REPORT QUESTION 7: Are there any noteworthy differences between the measured geometries of 3 with the compounds? Using torsion angles, comment on the relative orientations of the phenol and triazole rings in the two structures. What range of ring-twisting angles are observed for this set of three similar chemical structures? Why? Discuss the geometry that you measure with reference to standard values expected (e.g. from textbooks or reference works) for substituted aromatic and heterocyclic compounds.*

**NOTE**: Figure and data tabulation is similar to that of *REPORT QUESTION 2* & *5*

**

*REPORT QUESTION 8: Describe the hydrogen bonding structure observed. Consider the symmetry and shape of the observed structure. Compare and contrast this with compounds 1 & 2. What may help, is considering this compound is in what is called a space group labelled as P21/n.*

*In your answer, include a screenshot showing the hydrogen bonding structure/dimer and another screenshot with atoms labelled showing the “long way around” link as described in the additional Etter notation above.*

**Grading for average Exercise 3 (report questions 7-8): 93%**

# Appendix C: Survey Questions and Results

The following at the questions provided to the students for optional additional feedback. With 8 learners, 5 answered the survey and provided feedback:

Question 1:

I felt like this dry lab was accessible (with regards to data and software)

Strongly disagree (0), Disagree (1), Neutral (2), Agree (2), Strongly agree

Question 2:

I gained a better understanding of crystallography from this dry lab

Strongly disagree (0), Disagree (0), Neutral (2), Agree (3), Strongly agree (0)

Question 3:

This dry lab made me more curious about crystallography

Strongly disagree (0), Disagree (0), Neutral (0), Agree (4), Strongly agree (1)

Question 4:

I would like a longer lab involving crystallography

Strongly disagree (0), Disagree (1), Neutral (2), Agree (2), Strongly agree (0)

Question 5:

This dry lab complemented the associated wet lab

Strongly disagree (0), Disagree (1), Neutral (0), Agree (3), Strongly agree (1)

Question 6:

Is there any other feedback you wish to share?

(See summary in 3.1. Student Feedback)
